# Radiation Quality, Like Art, Consists in Drawing the Line Somewhere

**DOI:** 10.3389/fonc.2013.00163

**Published:** 2013-07-17

**Authors:** Charles A. Kunos, Ivy A. Petersen

**Affiliations:** ^1^Department of Radiation Oncology, Summa Health System, Akron, OH, USA; ^2^Department of Radiation Oncology, Mayo Clinic, Rochester, MN, USA

**Keywords:** radiation quality, radiation safety, radiochemotherapy clinical trial

## Abstract

The architects of phase I radiochemotherapy development programs impose a semblance of structured radiation “intensity” and adverse event predictability upon radiation-anticancer agent interactions whose natural complexity and improper mixing would otherwise lead to dire health consequences. It is incumbent upon radiation oncology investigators to pledge radiation quality and safety to the participants of radiochemotherapy trials. Measures of radiation quality and safety may be tools to scrutinize radiation-anticancer agent dose and schedule, as well as, radiation field design among diverse radiation delivery platforms. In this article, the merits and demerits of phase I radiochemotherapy quality and safety policies are critiqued considering the current era of rapidly evolving radiation technologies.

## Opinion

The traditional first-in-human radiochemotherapy study or phase I investigation often determines the dose and schedule of radiation and investigational anticancer agent combination. This assessment also paints the initial descriptive palette of adverse events linked with the co-administration of the two in a dose-dependent manner. From this parti, we gain an illustrative safety portfolio for radiation-anticancer agent combinations that allow next step study of therapeutic response in phase II or III clinical trials.

In an effort to maximize clinical benefit, architects of phase I radiochemotherapy development programs conduct stepwise dose-escalated trials of radiation, of anticancer agent(s), or both together. It has been held in such dose-escalated trials that one accepts an adverse event or toxicity rate of 33% or less in participants, based on the premise that the limited study subject numbers are largely representative of patient cancer groups sharing common traits. Discovery of a toxicity rate target may be done by escalating radiation or anticancer agent dose until two of six (i.e., 33%) enrollees manifest an adverse event, often a toxicity arising from a pre-determined list. For radiochemotherapy trials, investigators then drop down to the next lower radiation or anticancer agent dose level to enroll additional patients to establish a recommended phase II dose and schedule. This is coined the maximally tolerated dose (MTD). The end product is a radiation-anticancer agent combination recommended for phase II or III clinical testing. Here, we focus on stepwise dose-escalated trials but recognize that other radiation-drug discovery formats are part of the anticancer clinical development portfolio (Ivanova and Bunce, 2010).

Radiochemotherapy phase I trials are framed by three salient doctrines: patient wellbeing, principled conduct, and minimized interruption (Ivy et al., 2010). Indeed, a nod must be given to the courage of phase I enrollees to have medical treatment, on which their wellbeing may just depend, involving avant-garde treatment. Next follows intent to reduce the number of trial participants exposed to acute and lifelong serious, or even life-threatening, adverse events. To optimize efficiency, the least number of patients exposed to subtherapeutic radiation-agent doses merits serious and respectful scrutiny. When toxicity is used as a measure of radiochemotherapeutic activity and/or efficacy, responses in phase I radiochemotherapy studies may occur at a fraction of an investigational anticancer agent’s own active phase II dose and schedule. To illustrate this point in uterine cervix cancer treatment, gemcitabine given for radiosensitization [125 mg/m^2^ (Chavez-Blanco et al., 2005)] is a fraction (16%) of when gemcitabine is administered as a single agent [800 mg/m^2^ (Schilder et al., 2005)] or combined in a gemcitabine (800 mg/m^2^)-cisplatin (30 mg/m^2^) regimen (Brewer et al., 2006). As another example in uterine cervix cancer radiochemotherapy, much less (−75%) triapine (25 mg/m^2^) is administered for cisplatin radiochemosensitization (Kunos et al., 2010) than when used as a single agent [96 mg/m^2^ (Nutting et al., 2009)]. Close-knit phase I research teams are often able to standardize actual delivered radiation and anticancer agent dose and schedule as well as radiation treatment portal design.

But when it comes to large multi-site phase I trials, radiation therapy prescriptions and field designs can be inconsistent from patient-to-patient, perhaps affecting the toxicity profile of a radiation-anticancer agent combination. Take for instance the 19 patient study of two-cycle 5-fluorouracil (5FU, 1000 mg/m^2^/day over 96 h) and single-cycle bolus mitomycin C (15 mg/m^2^) radiochemotherapy (30 Gy in 15 2 Gy fractions) for anal cancer (Nigro et al., 1974, 1981; Buroker et al., 1977). In the first three patients, 5FU was administered 25 mg/kg body weight and mitomycin C was given 0.5 mg/kg body weight. No (0/3) substantial toxicity was reported (Nigro et al., 1974). In the next two treated patients, serious adverse events occurred. With a toxicity rate of 40% (2/5) (Nigro et al., 1981), chemotherapy dose was lowered for the next 14 treated patients. Reversible gastrointestinal toxicity was noted, but the investigators held to an 11% (2/19) overall toxicity rate (Nigro et al., 1981). A subsequent multi-site phase III trial of 5FU (1000 mg/m^2^/day over 96 h) plus mitomycin C (10 mg/m^2^, bolus day 1, 29) radiochemotherapy had planned adjustments in treatment intensity (Gunderson et al., 2012). Here, not only was the chemotherapy dose and schedule adjusted, but modifications in radiation delivery involved change in initial large pelvic field prescribed to 30.6 Gy in 17 1.8 Gy fractions, to a reduced field where the superior margin was lowered to the inferior level of the sacroiliac joints and prescribed to 14.4 Gy in eight 1.8 Gy fractions. A 10–14 Gy boost in five to seven 2 Gy fractions followed for T3, T4, N+, or T2 lesions with residual cancer after 45 Gy. After these revisions to the radiochemotherapy intensity, the study has resulted in a long-term rate of grade 3 or higher toxicity of 37% (119/325) for the 5FU/mitomycin C regimen (Gunderson et al., 2012). Essentially evolving from this study, a phase II trial of 5FU (1000 mg/m^2^ over 96 h, day 1, 29) and mitomycin C (10mg/m^2^, day 1, 29) radiochemotherapy investigated peer-reviewed dose-painted intensity modulated radiation therapy (IMRT) (Kachnic et al., 2013). The initial report has found that an IMRT technique resulted in a significantly reduced grade 3 or higher gastrointestinal toxicity rate of 21% (13/63) (Kachnic et al., 2013). Taken together, it appears that radiation technology as well as local investigator radiation portal design, perhaps guided by peer-review, sways observed radiochemotherapy adverse event rates.

What ought we to conclude from these clinical lessons when it comes to the design and the interpretation of phase I radiochemotherapy clinical trials? In this editorial, the merits and demerits of phase I radiochemotherapy quality and safety policies are appraised while mulling over the impact of rapidly evolving radiation technologies.

### Radiation dose and schedule

There can be little doubt that both radiation prescription and radiation dose conformity have an influence on the determination of tolerable radiation-anticancer agent combinations. To ensure safe and quality radiation in a phase I clinical trial program, it is essential that radiation dose prescription, treatment volume coverage, and normal as well as tumor tissue constraints be as uniform as possible.

From one viewpoint, radiation dose prescriptions have undergone careful stepwise evaluations in cancer-specific institutional trials and multi-center experiences to determine dose that sterilizes disease at tolerable levels of morbidity. Arbitrary modification of radiation prescription can unduly influence the appreciated collage of toxicity from radiation-anticancer agent combinations. One can appreciate that increasing radiation dose may lead to unanticipated observable toxicity that would adversely affect clinical judgment of whether a radiation-anticancer agent combination is tolerable. Likewise, decreasing radiation dose arbitrarily may result in an under appreciation of toxicity of the intended study regimen. Unapproved alterations in radiation dose intensity by participating investigators skews final assessments of whether radiation-anticancer agent combinations are safe. To encourage compliance, radiochemotherapy clinical trials usually include detailed radiation dose parameters to ensure target volume coverage built around minimum and maximum proportions of the nominal prescribed dose.

Typically, radiation dose conformity across target volumes should be as homogenous as possible relative to a recommended prescription dose. In practical terms for dose, overseers of radiochemotherapy trials rely on treatment plan normalization points, which can be left to the investigator’s preference and really only guides display of isodose contours, when assessing radiation quality. It is recognized that differences between calculation algorithms vary among planning systems up to 30% in extreme cases (Schuring and Hurkmans, 2008). But one can certainly appreciate that careful monitoring of the placement of the normalization point, disease location, and overall design of the radiation plan influence radiation quality. So too, radiation machine output factors into radiation quality. Quality assurance programs, including those of phase I radiation centers, have built in daily checks and balances to ensure accurate radiation dose delivery. As such, variance in machine output should be minimal, with tolerance ranging from plus or minus 3%. When it comes to multi-center phase I radiochemotherapy clinical trials, these tools of radiation quality (i.e., radiation dose conformity, radiation machine output checks) are monitored frequently to ensure best practice and style. Logistically, this conjures up cohesive and centralized teams of physicians and radiation physicists to ensure utmost care in radiation delivery among radiochemotherapy trial enrollees.

Protracted delay in treatment schedule modifies assessment of “risk-adapted” radiation dose. Take for instance a radiation schedule involving daily external beam radiation and co-administration of an investigational anticancer agent that is also integrated with ablative brachytherapy. Single external beam treatment day missed appointments may have minimal impact on overall intensity of radiation-anticancer agent administration. But multiple day or multiple week deviations from expected radiation-anticancer agent courses modifies the radiobiological effect on normal and cancer cells. Since patient safety is of primary concern in phase I studies, normal tissue cell recovery and renewal occurring because of protracted radiation dose delivery could lessen apparent toxicity. Such circumstances may be misleading by under representing toxicity which would have been witnessed at the intended dose and schedule.

### Continual radiation quality reassessment

It seems prudent to provide rapid assessment and reassessment of radiation delivery under phase I clinical trial conditions. Phase I radiochemotherapy programs are moving toward timely radiation review prior to turning on the first treatment beam. Computer-aided plan review and teleconferencing allows near real-time centralized critique and assurance that radiation protocol guidelines are met. Any discrepancies can be discussed, arbitrated, and accepted by the treating radiation oncologist and by the reviewing radiation principal investigator or designee. It does seem sensible to have at least two unbiased radiation oncologists review and discuss radiation quality of cases in a phase I program. Rendered decisions can be communicated to the treating radiation oncologist and sponsors a culture of on-going learning. Ideally, as new phase I radiochemotherapy studies develop from prior experience in a disease, a core group of expert radiation oncologists could provide longitudinal consistency across the radiation treatment plans and delivery.

### Radiation field and/or volume design

#### Radiation safety has rarely been more demanded – or more newsworthy

For the past 5 years, the American Society for Radiation Oncology has promoted recommendations as to how radiation therapy could be made safer and higher quality. Adverse radiation safety events (van Sickler, 2005) hurt radiotherapy’s reputation – and create a dilemma for many early phase radiochemotherapy investigators who rely on the courage of patients to enroll in their clinical trials. In radiochemotherapy trials, the incidence of toxic events often parallels radiation treatment portal size, so when portal size is large, toxicities increase. After an era of two- and three-dimensional radiation therapy planning involving sizeable radiation portals intending gross and occult disease, even small enlargements in the treatment portal produce more frequent adverse toxicity events.

Pelvic radiation fields are especially vulnerable. A standardized four-field box pelvic radiation field expanded in all dimensions by 1 cm can expose up to an added 60% volume of irradiated tissue. Such a field design modification may substantially elevate total irradiated volume of bowel and bone marrow, certainly to impact adverse event rates in these tissues. Other seemingly safe treatment field modifications are at risk, too. In uterine cervix cancer radiation treatment, a parametrial boost field may be used in clinical situations that call for higher dose to the pelvic sidewalls and pelvic lymph nodes. Even though a parametrial boost field adds 5.4–9.0 Gy of radiation to the typical 45 Gy of four-field radiation, investigators must be wary of the rising radiation-related risks to bowel. For instance, keeping a superior border of the standardized parametrial boost field at a L4/L5 vertebral body junction and not lowering the border to 1 cm above the inferior margin of the sacroiliac joint increases the irradiated abdominopelvic volume by nearly 130%. Since small bowel is more radiosensitive than other pelvic structures manifests a variety of symptoms that register as adverse events, and impacts quality of life in the short and long-term, any increases in radiation-related exposure could tip the balance in determining whether a radiation-anticancer agent combination is safe.

It is difficult to muster much enthusiasm for greater scrutiny of investigator individualized radiation treatment portal design. Yet, radiochemotherapy phase I overseers would be unwise to flee from offering such scrutiny. The tiniest of deviations away from standard radiation treatment portal design can have significant influence over adverse events noted on a clinical trial. The rate of adverse events directly feeds into the decision making process of whether a novel radiation-anticancer agent combination is safe and worthwhile for further testing. That is because small patient phase I trials serve not only as a source of critical early development data but also buffer the volatility of the ever-changing interests of early phase radiation-anticancer agent drug programs. In an era when many diseases are treated with a multimodality approach, it is vital to control all factors in the delivery of that treatment in order to ensure appropriate analysis of these new agents.

But there are ways for investigators to manage “risk-adapted” phase I radiochemotherapy trials. A first step is to standardize quality radiation-anticancer agent delivery. Then, as a second step, investigators should explore radiation technologies that have better prospects for normal tissue tolerance to radiation exposure. The advent of IMRT, helical tomotherapy, and arc-based or robotic stereotactic ablative radiosurgery (SABR) has brought new challenges for assurance of radiation quality and safety. These technologies along with adaptive radiotherapy and image guided radiotherapy (IGRT) rely on treatment volume definitions which are still in development in many disease sites. In radiochemotherapy centers and cooperative groups known for excellence in the quality delivery of radiation, rapid review of cases allows for uniform definition of radiation volumes and dose delivered over elapsed times specified in clinical trial protocols with little variation. Real-time monitoring of these advanced radiation delivery platforms merits our serious and respectful scrutiny. Treatment volumes are not as easily defined by bony anatomy as is seen in 2 and 3-D treatment volumes and can be variable depending on differing patient anatomy. Hence these platforms should not earn our uncritical acceptance. Arguably, these platforms offer expectantly substantial reduction in radiation-related adverse events but should not be used clinically in deference to rigorous quality assurance by all users. Also, it is prudent within a single phase I radiochemotherapy trial to have a uniform radiation platform by all investigators. Enrolling trial participants into a phase I radiochemotherapy trial that allows radiation treatment by conventional radiation or by IMRT would cloud the interpretation of observed radiation-drug adverse events, possibly raising issues of falsely concluding a safe relationship exists when it truly may not. Mixing technologies on a phase I radiochemotherapy trial may also invalidate the historical radiation-specific technology adverse event data upon which the trial was predicated. Observed toxicity on down-the-line phase II or phase III studies may also be influenced by “any” form radiation delivery. More study is needed and guidelines for safety and quality assurance are emerging for these new technologies and utilization in phase I radiochemotherapy studies should only be considered once these guidelines are confirmed in other venues.

## Conclusion

Making radiation-anticancer agent discovery safer and of optimal quality is the goal of phase I radiochemotherapy programs. Implementing the layers of quality control such as dose checks, timetables, uniformity in treatment portal/treatment volume design, and brachytherapy application, and with timely critical feedback are musts in high-performing phase I radiochemotherapy programs. In the context of radiochemotherapy, the architects of clinical trials must layout plans intended to balance the safe selection and concise use of radiation-anticancer agent combinations.

## Conflict of Interest Statement

The authors declare that the research was conducted in the absence of any commercial or financial relationships that could be construed as a potential conflict of interest.
